# Electrochemical reactivity of urea at Pt(100) surface in 0.5 M H_2_SO_4_ by AC impedance spectroscopy

**DOI:** 10.1007/s10008-012-1936-8

**Published:** 2012-11-17

**Authors:** Boguslaw Pierozynski

**Affiliations:** Department of Chemistry, Faculty of Environmental Protection and Agriculture, University of Warmia and Mazury in Olsztyn, Plac Lodzki 4, 10-957 Olsztyn, Poland

**Keywords:** Urea (U), Guanidinium cation (G^+^), Pt(100) single crystal, Impedance spectroscopy

## Abstract

The present paper reports an alternate current impedance spectroscopic study on adsorption of urea (U) at Pt(100) single-crystal surface, examined in 0.5 M H_2_SO_4_ supporting electrolyte. The resulted information provided confirmation of the role of electrosorption of urea on the Pt(100) plane through evaluation of the associated charge transfer resistance and capacitance parameters. Obtained impedance results were compared to those previously recorded for guanidinium cation (G^+^) under analogous experimental conditions, especially with respect to the so-called *ion pairing* mechanism, as originally proposed for the G^+^ ion and bi(sulfate)/OH species, based on the voltammetric and in situ Fourier transform infrared spectroscopy results.

## Introduction

Some earlier works from this laboratory were concerned with electrosorption and electroreactivity of small organic molecules at well-ordered Pt single-crystal surfaces, including: aliphatic oximes [1, 2] and guanidinium [G^+^/^+^NH_2_ = C(NH_2_)_2_]-type ions [3–5]. With respect to G^+^ ions, their presence in solution was revealed in substantial effects on the voltammetric profiles for UPD of H, both in acidic and in alkaline media, owing to two-dimensional interaction effects between the adsorbed organic cations and electrosorbed bi(sulfate) (or OH^−^) species. This phenomenon was originally [3] termed *ion pairing* or *cooperative chemisorption*. These effects were also characterized in detail by in situ Fourier transform infrared spectroscopy (FTIR) experiments at Pt(111) and (100) planes [4], as well as by alternate current (AC) impedance spectroscopy kinetic investigations, carried out in 0.5 M H_2_SO_4_ solution at the (100) surface [5].

Urea [O = C(NH_2_)_2_] is a structure-related molecule to that of guanidine. Its adsorption behavior at Pt single crystals was widely studied in the past. Hence, Climent et al. in Refs. [6] and [7] examined in situ FTIR and cyclic voltammetry behavior of urea in 0.1 M HClO_4_ at Pt(100) and (111) planes, respectively. Urea was shown there to undergo dissociative electrosorption on the surface of Pt in an anodic (1- or 2-electron transfer) oxidation process [6, 7]. Similar cyclic voltammetry experiments on adsorption of urea in HClO_4_ at various low-index Pt surfaces were reported by Rubel et al. [8]. In addition, important radiochemistry measurements [9, 10] were conducted in order to facilitate derivation of the potential and concentration dependence of urea coverage on Pt, including the surface structure for the urea adlayer on Pt(100) plane [10].

The key aim of this work was to present the kinetic aspects of the process of electrosorption of urea on the Pt(100) plane in 0.5 M H_2_SO_4_, especially in reference to those recently reported for guanidinium cations under analogous experimental conditions. The above was accomplished through derivation of the Faradaic resistance and electrosorption pseudocapacitance components associated with UPD of H and electrosorption of U, carried-out at several working electrode potentials.

## Experimental

Pt single-crystal of the (100) orientation was prepared from 1 mm diameter 99.9985 % Pt wire (AESAR/Puratronic) by employing the techniques and procedures for preparation of Pt single crystals developed by Clavilier et al. [11]. High-purity, aqueous 0.5 M H_2_SO_4_ solution was prepared from sulfuric acid of highest purity available (SEASTAR Chemicals) with water derived from an 18.2 MΩ Direct-Q3 UV ultrapure water system from Millipore. Urea (Stanlab, pure, p.a., Poland) was used to prepare acidic solutions, at a concentration of 1 × 10^−3^ M U. All prepared solutions were de-aerated with high-purity argon (Ar 6.0 grade, Linde), which was also purged above the solutions during impedance measurements.

AC impedance measurements were conducted by means of the *Solartron* 12,608 W Full Electrochemical System, consisting of 1,260 frequency response analyzer (FRA) and 1,287 electrochemical interface. All potential measurements were referred to the potential of a reversible hydrogen electrode (RHE), in the same test solution. The 1,260 FRA generator provided an output signal of known amplitude (5 mV) and the frequency range was usually swept between 1.0 × 10^5^ and 0.1 Hz. The instruments were controlled by *ZPlot 2*.*9* software for Windows (Scribner Associates, Inc.). Presented impedance results were obtained through selection and analysis of representative series of experimental data. Usually, three impedance measurements were carried out at each potential value. Reproducibility of such-obtained results was typically below 10 % from one measurement to another. The impedance data analysis was performed with *ZView 2*.*9* software package, where the spectra were fitted by means of a complex, nonlinear, least-squares immitance fitting program, *LEVM 6*, written by Macdonald [12]. An equivalent circuit employed to analyze the obtained impedance results is later shown in Fig. 3.

## Results and discussion

### Cyclic voltammetry behavior of urea at Pt(100) single-crystal plane in 0.5 M H_2_SO_4_ solution

**Fig. 1:**
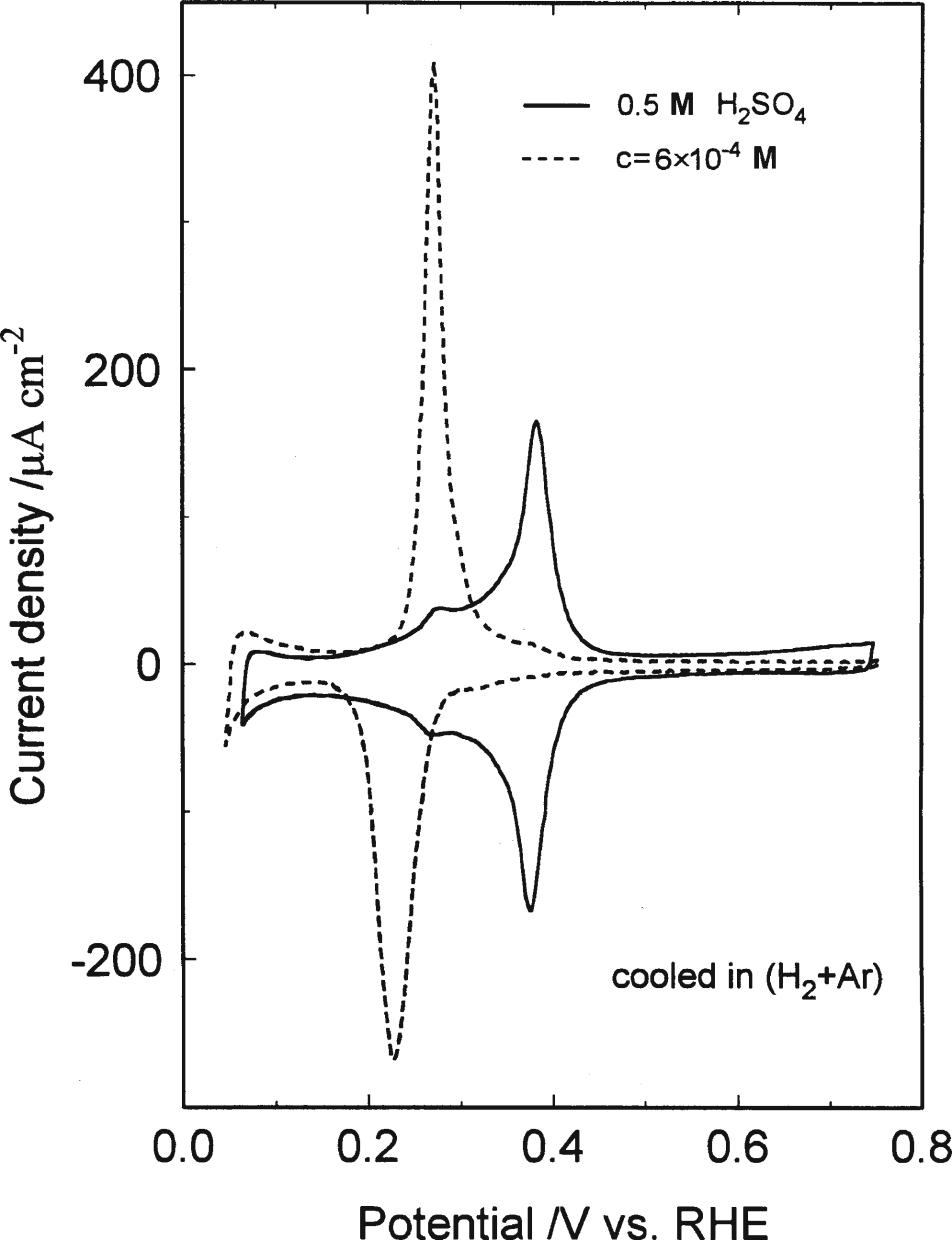
Cyclic voltammograms for Pt(100) in 0.5 M H_2_SO_4_ recorded at a sweep rate of 50 mV s^−1^ and in the presence of U at the concentration indicated; voltammograms were recorded on the third cycle (from Ref. [4])

Figure 1 below presents the adsorption behavior of urea [4] (at a concentration of 6 × 10^−4^ M U) at the Pt(100) plane in 0.5 M H_2_SO_4_. The adsorption effects of U are characterized by the potential range for UPD of H being “squeezed” and considerably (by *ca*. 150 mV) shifted in a negative direction. In this respect, the voltammetric behavior of urea is analogous to that previously reported for guanidinium ions at the Pt(100) surface [3, 4]. These effects (see Fig. 1) could conveniently be explained in terms of attractive, *ion pairing* interactions between co-adsorbed urea molecules and HSO_4_^−^ ions (see Structure 1 below) thus referring to those previously examined for the G^+^ ion by means of cyclic voltammetry and FTIR measurements in Refs. [3] and [4].

**Fig. 2:**
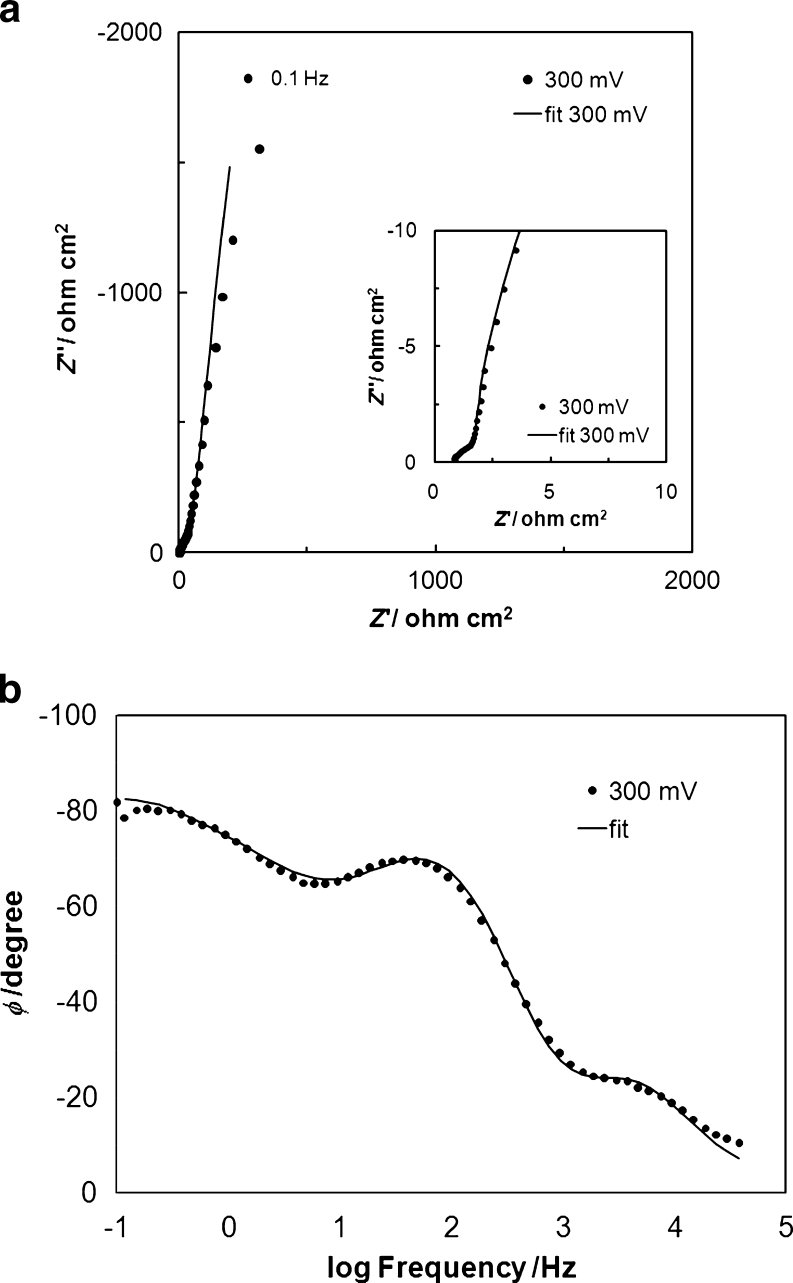
**a** Complex plane impedance plot for Pt(100) in contact with 0.5 M H_2_SO_4_ in the presence of 1 × 10^−3^ M U recorded at 293 K for the stated potential value. The *solid line* corresponds to representation of the data according to the equivalent circuit shown in Fig. 3. **b** Bode phase-angle plot for impedance behavior at Pt(100) in contact with 0.5 M H_2_SO_4_ in the presence of 1 × 10^−3^ M U, for the stated potential value (other details as in Fig. 2a)

Although at the Pt(100) plane, the recorded [4] voltammetric behavior of U was comparable with that observed for the G^+^ ions, on the (111) surface, urea was suggested [4] to exhibit significantly reduced *ion pairing* interactions with bi(sulfate) species than the corresponding guanidinium ions. However, it has to be stressed that it is predominantly sulfate (*not* bisulfate) species that becomes adsorbed on the Pt(111) plane in sulfuric acid solution (see recent works by Su et al. [13], by Garcia-Araez et al. [14], and by Yeh et al. [15]). Thus, in this case, no attractive interactions between the adsorbed, unprotonated urea molecules and sulfate species could be envisaged [contrast to the behavior at the (100) plane, see Structure 1 again]. Consequently, for the (111) plane, the recorded displacement of the voltammetric profile in the presence of urea (see Fig. 7a in Ref. [4]) is most likely the result of partial protonation of urea in the supporting electrolyte (please note that about 10 % of U molecules at pH ≈ 1 exist in a cationic form [3, 4, 6]). The above-made conclusions get support from the previously performed [4] FTIR experiments in the presence of urea, which indicated considerably less extensive chemisorption of U on the Pt(111) surface, as compared to that exhibited at the (100) plane.

### AC impedance behavior of urea at Pt(100) plane in 0.5 M H_2_SO_4_ solution

The impedance behavior of urea, at a concentration of 1 × 10^−3^ M U in 0.5 M H_2_SO_4_ at the Pt(100) plane is shown in Fig. 2a and b and in Table 1. Thus, the impedance behavior of U at potentials close to that of the capacitive peak in Fig. 1 (200, 250, 300, and 350 mV vs. RHE) is characterized by the appearance of two partial semicircles and a capacitive line at an inclination to the *Z*′ axis different from 90°. The smaller semicircle (see the impedance spectrum recorded at 300 mV in inset to Fig. 2a), observed at high frequencies, corresponds to the process of UPD of H (in relation to the charge transfer resistance, *R*_H_), and the part of a large-diameter semicircle (see Fig. 2a again), observed throughout the intermediate frequency range, is associated with the charge transfer process (with respect to the *R*_U_ parameter in Table 1) accompanying electrosorption of urea on the Pt surface (see Eq. 1 below [7]). Moreover, existence of two maxima is clearly discernible in the corresponding Bode phase-angle plot in Fig. 2b (please evaluate the fitting quality by the derived chi-squared: χ^2^ parameter values given in Table 1).

**Fig. 3:**
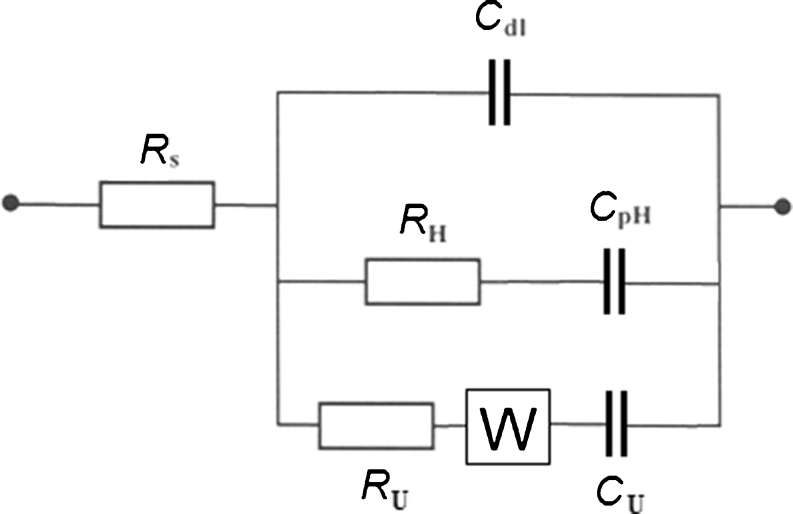
Equivalent circuit for an adsorption process such as UPD of H, exhibiting Faradaic pseudocapacitance, *C*_pH_, charged via a Faradaic resistance, *R*_H_ in the presence of co-adsorbed urea (*R*_U_ and *C*_U_ components), in a parallel combination with the double-layer capacitance, *C*_dl_, jointly in series with an uncompensated solution resistance, *R*_S_; *W* diffusional Warburg element, employed as an empirical term

**Table 1:** Resistance and capacitance parameters for UPD of H and the process of electrosorption of U molecule (at a concentration of 1 × 10^−3^ M) on Pt(100) plane in 0.5 M H_2_SO_4_ (recorded at 293 K), obtained by finding the equivalent circuit which best fitted the impedance data, as shown in Fig. 3 (*χ* ^2^ refers to the recorded chi-squared parameter values by the *ZView* fitting software)

*E*/mV	*R*_H_/Ω cm^2^	×10^6^ *C*_pH_/F cm^−2^	×10^6^ *C*_dl_/F cm^−2^	*R*_U_/Ω cm^2^	×10^6^ *C*_U_/F cm^−2^	χ^2^
200	0.45 ± 0.01	395.7 ± 3.3	28.5 ± 1.7	267.0 ± 6.5	304.5 ± 6.3	4.7 × 10^−4^
250	0.55 ± 0.02	378.8 ± 3.8	36.5 ± 1.9	133.1 ± 5.6	2,089 ± 95	9.4 × 10^−4^
300	1.85 ± 0.03	159.4 ± 1.7	43.2 ± 1.8	148.8 ± 3.2	291.4 ± 5.5	5.6 × 10^−4^
350	4.70 ± 0.22	61.8 ± 2.0	55.4 ± 2.2	513.8 ± 12.6	205.2 ± 3.9	7.2 × 10^−4^

As compared to the case of pure 0.5 M H_2_SO_4_ supporting electrolyte (e.g., see Table 1 in Ref. [5]), the kinetics of the process of UPD of H have become significantly slowed down in the presence of U, beyond the potential of 250 mV vs. RHE (Table 1). A significant increase of the *R*_H_ parameter (from 0.45 Ω cm^2^ at 200 mV to 4.70 Ω cm^2^ at 350 mV) can be explained in terms of the *ion pairing* mechanism (originally proposed for the G^+^ ions in Ref. [3]), where the presence of surface-adsorbed bisulfate species, arising at significantly lower electrode potentials together with co-adsorbed U molecules (Structure 1), appreciably influences the kinetics of UPD of H on this Pt plane. In addition, changes of the hydrogen adsorption capacitance (*C*_pH_) strictly follow those of the *R*_H_ parameter; thus, the *C*_pH_ dramatically declines (Table 1) from 395.7 μF cm^−2^ (at 200 mV) to 61.8 μF cm^−2^ (at 350 mV).1


On the other hand, the adsorption charge transfer resistance for urea (*R*_U_) reaches its minimum value of 133.1 Ω cm^2^ at the potential close to that of the peak current–density in Fig. 1 (250 mV), which implies that the kinetics of adsorption of U on the Pt(100) plane are dramatically slower than those of UPD of H on this surface. Again, minimum of the *R*_U_ value coincides with a very large value (2,089 μF cm^−2^) of the adsorption pseudo-capacitance parameter (*C*_U_), recorded at the potential of 250 mV.

Furthermore, the recorded double-layer capacitance values (*C*_dl_) in Table 1 oscillate between *ca*. 28 and 55 μF cm^−2^. These *C*_dl_ values are considerably higher than 20 μF cm^−2^, i.e., a commonly quoted double-layer capacitance value in literature for smooth and homogeneous surfaces [16, 17], which implies some contribution to the recorded *C*_dl_ from the adsorption capacitance components (Table 1). Moreover, an observed deviation from the purely capacitive, 90° phase angle behavior (also expressed by “depressed” semicircles in the Nyquist impedance plots, see Fig. 2a) corresponds to dispersion of capacitance. The phenomenon of capacitance dispersion is typically visualized as corresponding either to slow adsorption/desorption processes or as the effect of increasing Pt surface inhomogeneity, especially important when extensive potentiostatic impedance measurements take place [18–20]. All fittings of the recorded impedance data were performed by means of the double-adsorbate equivalent circuit (see Fig. 3) with employment of a Warburg diffusional element (*W*), regarded here as an empirical term [21] involved in the kinetics of urea electrosorption on the Pt(100) plane.

**Fig. 4:**
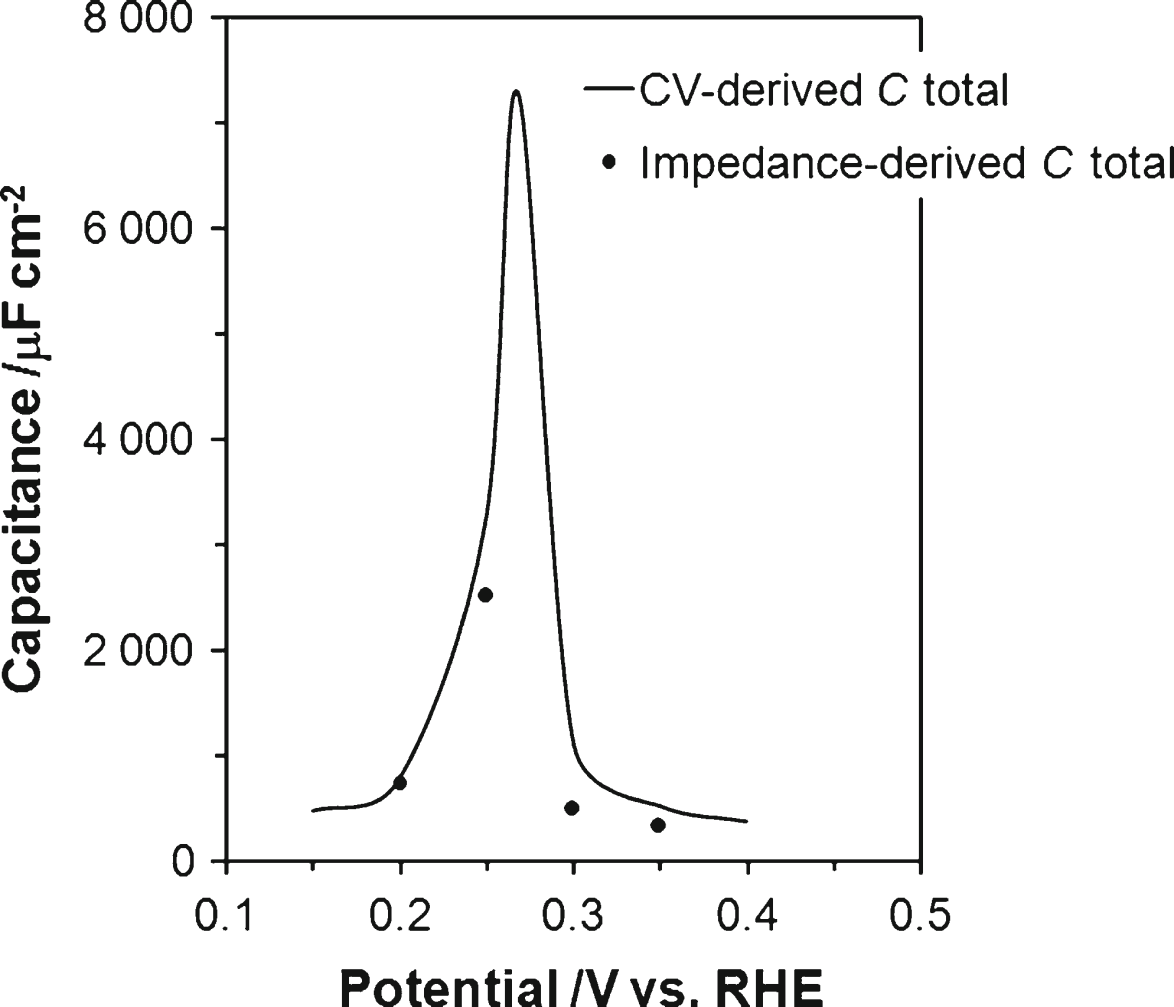
Total capacitance (*C*_total_) as a function of the applied potential (vs. RHE) for the desorption of H and adsorption of U (at a concentration of 1 × 10^−3^ M) on Pt(100), in contact with 0.5 M H_2_SO_4_ solution. The solid line corresponds to the *C*_total_ calculated from the CV profile (*C*_total_ = current–density/sweep rate) and the experimental points are the impedance-derived values of total capacitance

As an internal check on the impedance results, Fig. 4 below shows total capacitance, *C*_total_, where *C*_total_ = *C*_dl_ + *C*_p_ (adsorption pseudocapacitance components), as a function of potential for the adsorption behavior of U on the Pt(100) single-crystal surface. This figure compares *C*_total_ obtained from the impedance results with that directly calculated from the voltammetric profile [5, 22] under comparable experimental conditions (1 × 10^−3^ M U). It can be seen in Fig. 4 that the *C*_total_ values are in fairly good agreement with each other for all four “probing” potential values.

**Structure 1:**
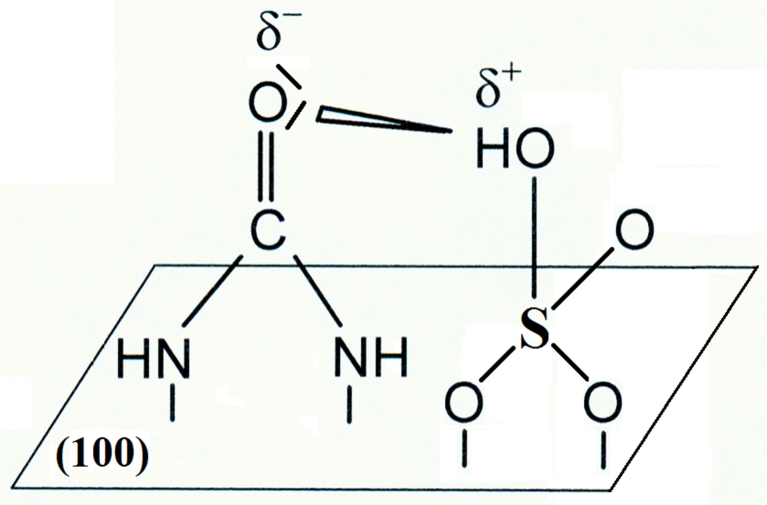
Ion pairing interactions between co-adsorbed urea molecules and HSO_4_^−^ ions

With respect to the impedance behavior previously recorded in the presence of the guanidinium cation [5], the process of electrosorption of urea at the Pt(100) plane is characterized by significantly increased values of the charge transfer resistance parameter (*R*_U_ vs. *R*_G+_ in Ref. [5]). In fact, the *R*_U_/*R*_G+_ ratio comes to *ca*. 5.6 for the minimum electrosorption resistance values, recorded for U and G^+^ species, respectively. The above strongly supports the formerly derived conclusions [4] on the *ion pairing* (dipole–dipole type) between the electrosorbed U molecules and bisulfate species being significantly weaker than the corresponding G^+^/HSO_4_^−^ (ion dipole) interactions (not particularly evident from the comparative: U vs. G^+^ cyclic voltammetry behavior for this Pt plane). In this respect, the electrochemical behavior of urea on the Pt(100) plane is somewhat analogous to that recorded for *N*,*N*-dimethyl-guanidinium (DMG^+^) cation in Ref. [5].

## Conclusions

Application of AC impedance spectroscopy to study adsorption behavior of urea at Pt(100) single-crystal surface provided support for the cooperative: U/HSO_4_^−^ electrosorption mechanism for U (also with respect to significance of UPD of H), based on the impedance-derived charge transfer resistance and capacitance components.

Moreover, significantly increased values of the adsorption charge-transfer resistance for urea (with respect to those previously recorded for guanidinium ion at Pt(100) plane in Ref. [5]) suggest considerably slower electrosorption of urea (and respectively weakened U/HSO_4_^−^ *ion pairing*) than that previously reported for G^+^ on this Pt plane.

## References

[1] Pierozynski B, Conway BE (2002). J Electroanal Chem.

[2] Pierozynski B, Kowalski IM (2011). J Electroanal Chem.

[3] Pierozynski B, Morin S, Conway BE (1999). J Electroanal Chem.

[4] Pierozynski B, Zolfaghari A, Conway BE (2001). Phys Chem Chem Phys.

[5] Conway BE, Pierozynski B (2008). J Electroanal Chem.

[6] Climent V, Rodes A, Orts JM, Feliu JM, Perez JM, Aldaz A (1997). Langmuir.

[7] Climent V, Rodes A, Orts JM, Aldaz A, Feliu JM (1999). J Electroanal Chem.

[8] Rubel M, Rhee CK, Wieckowski A, Rikvold PA (1991). J Electroanal Chem.

[9] Horanyi G, Inzelt G, Rizmayer EM (1979). J Electroanal Chem.

[10] Gamboa-Aldeco M, Mrozek P, Rhee CK, Wieckowski A, Rikvold PA, Wang Q (1993). Surf Sci.

[11] Clavilier J, Pineaux R (1965). C R Acad Sci Paris.

[12] Macdonald JR (1990). Electrochim Acta.

[13] Su Z, Climent V, Leitch J, Zamlynny V, Feliu JM, Lipkowski J (2010). Phys Chem Chem Phys.

[14] Garcia-Araez N, Climent V, Rodriguez P, Feliu JM (2010). Langmuir.

[15] Yeh KY, Restaino NA, Esopi MR, Maranas JK, Janik MJ (2012) Catal Today. doi:10.1016/j.cattod.2012.03.011

[16] Lasia A, Rami A (1992). J Applied Electrochem.

[17] Chen L, Lasia A (1991). J Electrochem Soc.

[18] Pajkossy T (1994). J Electroanal Chem.

[19] Conway B, Barsoukov E, Macdonald JR (2005). Impedance behavior of electrochemical supercapacitors and porous electrodes. Impedance spectroscopy. Theory, experiment, and applications.

[20] Pell WG, Zolfaghari A, Conway BE (2002). J Electroanal Chem.

[21] Pajkossy T, Kolb DM (2009). Electrochim Acta.

[22] Morin S, Dumont H, Conway BE (1996). J Electroanal Chem.

